# A Survey of Knowledge, Attitudes, and Practices in Relation to Mosquitoes and Mosquito-Borne Disease in Western Australia

**DOI:** 10.3389/fpubh.2016.00032

**Published:** 2016-02-29

**Authors:** Abbey Potter, Andrew Jardine, Peter J. Neville

**Affiliations:** ^1^Medical Entomology, Environmental Health Hazards Unit, Department of Health, Perth, WA, Australia; ^2^School of Pathology and Laboratory Medicine, The University of Western Australia, Crawley, WA, Australia; ^3^Medical and Health Sciences, Edith Cowan University, Joondalup, WA, Australia

**Keywords:** arbovirus(es), mosquito(es), KAP survey, public health, vector-borne diseases

## Abstract

On average, more than 1,000 individuals will acquire a mosquito-borne disease in Western Australia (WA) each year. Knowledge, attitudes, and practices (KAP) in relation to mosquitoes and mosquito-borne disease have not yet been investigated within Australia. A randomized telephone survey of 2,500 households across 12 regions in WA was undertaken between February and May 2014. The aim of the survey was to obtain baseline KAP data surrounding mosquitoes and mosquito-borne diseases in different regions of WA, across a range of age groups and between males and females. The results of this survey indicate that the majority of respondents are aware of the potential for mosquitoes in WA to transmit *Ross River virus*, while awareness of other endemic mosquito-borne diseases remains limited. Common misconceptions exist in relation to exotic mosquito-borne diseases, with respondents incorrectly identifying malaria and dengue as endemic diseases in WA. The survey also highlighted a range of important issues, such as limited awareness of the potential for backyard breeding in domestic containers, occupational exposure to mosquitoes in regions with a large employment base in the mining and resources sector, increased exposure to mosquitoes as a result of participation in outdoor recreational activities in the north of the State, and reduced awareness of mosquito-borne disease in individuals aged 18–34 years. The results of this study will be used to inform the development of a new communication strategy by the Department of Health, to further raise awareness of mosquito-borne disease in WA. The data will then provide a baseline against which to compare future survey results, facilitating the rigorous evaluation of new communication efforts.

## Introduction

Each year, more than one billion people are infected and more than one million die from a mosquito-borne disease (1). In Western Australia (WA), individuals are at risk of acquiring the debilitating diseases caused by *Ross River virus* (RRV), *Barmah Forest virus* (BFV), *West Nile virus* strain Kunjin (WNV_KUN_), and the potentially fatal *Murray Valley encephalitis* (MVE) virus. RRV (Togaviridae: *Alphavirus*) is the most common mosquito-borne pathogen in WA, causing fever, rash, arthralgia, and myalgia in clinically affected people (2–4). Disease caused by BFV presents similarly to RRV, although symptoms are typically less severe (5). MVE is a rare but potentially fatal disease associated with a range of neurological signs, occurring in the northern half of WA (6). WNV_KUN_ is much less common, with only five cases of the disease being reported in WA since 2000, the last occurring in 2006 (7).

The WA Department of Health currently issues timely media warnings in response to the detection of viral activity, extreme weather events or increased mosquito abundance in an attempt to raise awareness of the increased risk of mosquito-borne disease transmission. The Department also provides information to the general public through pamphlet distribution on request, website information, and by supporting local government publicity campaigns. These efforts are considered largely passive. The promotion of important health messages in relation to mosquito-borne diseases in WA may be enhanced through more active communication channels.

As a first step toward developing a new communication strategy, a quantitative survey of the current knowledge, attitudes, and practices (KAP) in relation to mosquitoes and mosquito-borne disease in WA was undertaken. Survey questions surrounding knowledge of mosquitoes and mosquito-borne disease will help the Department to identify where efforts need to be focused in the future. Questions surrounding attitudes to various mosquito-related topics will provide an insight into why individuals hold particular viewpoints and help to inform efforts to drive changes in attitude, where necessary (8). Finally, questions surrounding practices will provide valuable information on the behaviors of individuals in relation to mosquito avoidance that may increase or decrease their risk of being bitten by mosquitoes.

This is the first-time a large-scale KAP survey of mosquitoes and mosquito-borne disease has been undertaken in WA. The data obtained from this study will assist in identifying gaps in knowledge, attitudes, and practices among regional, age, and gender groups that may hinder public health efforts to reduce mosquito-borne disease. The Department will use the results of this study to develop a new communication strategy targeting risk groups identified in the survey. Through repeated KAP surveys in the future, the data from this study will then be used to provide a baseline against which to rigorously evaluate its efficacy.

## Materials and Methods

### Study Area

**Figure 1:**
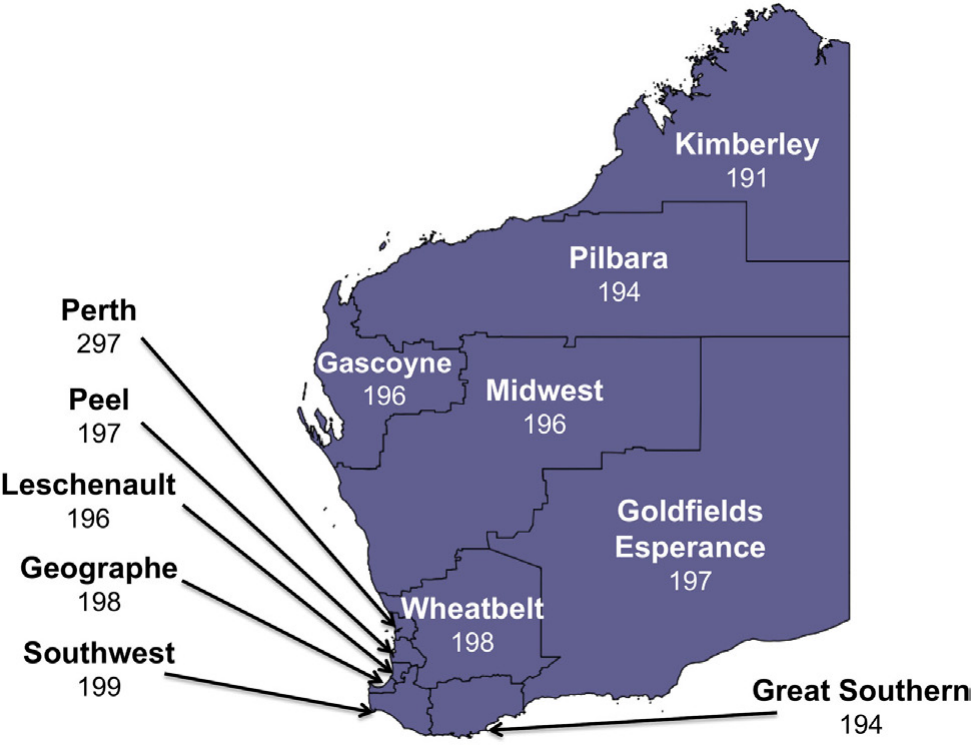
**The number of survey respondents from each region in Western Australia (total: 2453)**.

Western Australia occupies the entire western third of Australia, with a total land area of 25,269,875 km^2^ (9). The state has over 2.5 million inhabitants, with the majority residing in the southwest corner of the state (10). In total, 12 geographical regions were surveyed in this study, including Metropolitan Perth, Gascoyne, Goldfields-Esperance, Great Southern, Kimberley, Midwest, Pilbara, Southwest, and Wheatbelt regions. The Southwest was further divided into Peel, Geographe, Leschenault, and Southwest (other) to permit a more detailed analysis of this populated and high risk mosquito-borne disease region (Figure 1).

### Survey Development and Data Collection

A KAP survey was used to capture representative data on the knowledge, attitudes, and practices of individuals in relation to mosquitoes and mosquito-borne disease in WA. The survey was developed by the Medical Entomology branch within the WA Department of Health. Assistance in survey design was received from the Department’s Epidemiology team. The survey was conducted by the Survey Research Centre (SRC) at Edith Cowan University. The SRC was employed to undertake a computer-assisted telephone interview (CATI) survey by professionally trained interviewers using a stratified random process to capture households across all regions of WA. The survey instrument included a total of 34 questions regarding general knowledge, attitudes, and practices regarding mosquitoes and mosquito-borne disease, and included a mixture of open and closed questions. For closed questions in which survey participants were asked to select the most relevant response from a range of options, the order in which the options were read out were randomized.

Given the highly uneven population distribution in WA with over three quarters of the State’s population located in the Perth metropolitan area (11), all other regions were oversampled in order to obtain reliable estimates. Final sample sizes of *n* = 300 for the Perth metropolitan region and *n* = 200 from each of the remaining 11 regional divisions were surveyed. Households were selected at random by postcode using the electronic white pages listing landline telephone numbers in WA. Respondents were chosen at random from a pool of 44,326 households. A pilot survey of 27 individuals was undertaken to ensure the questions elicited an appropriate response, and there were no problems with the entry of answers into the database. Due to the difficulty with obtaining male respondents, the interviewer first asked to speak to any male over the age of 18 years. If no male respondent was available, the interviewer then asked to speak to the person with the next birthday in the household. The interviewer made three attempts to ring back and speak to the selected individual, before replacing them with another.

The SRC are permitted to conduct surveys with respondents over the age of 18 years without specific ethics approval, given the study is a general population survey, the topic is not sensitive, no individual identifying information is supplied and the samples are drawn from the electronic white pages. This study fulfilled all of the aforementioned conditions.

### Data Analysis

In order to facilitate comparisons between regions, obtain representative estimates for WA as a whole and compensate for the over-sampling in the regions outside Perth, the survey data were weighted by age, sex, and region to the 2013 estimated resident population (12). Data are presented in tables with survey locations listed from north to south to make it simpler to visualize and compare regional differences.

In some instances, respondents replied to questions with a descriptive answer that the surveyor recorded in an “other” or “additional information” option. Following the completion of the survey, each descriptive answer was assessed and where appropriate, the response was recoded against a pre-coded option if one existed. For example, when asked to name a mosquito-borne disease locally acquired in WA, individuals commonly answered “encephalitis” or “northern encephalitis.” These responses were recoded as MVE, as the disease is commonly known by shorter versions of the name.

Differences between regions, age groups, and sexes were defined as statistically significant if the 95% confidence intervals (CIs) of two estimates did not overlap. It should be noted that this is a conservative test of significance that reduces the significance level for each individual association. In comparison to statistical methods that use the more common significance level of 0.05, this method requires a larger difference between two estimates for the finding to be considered statistically significant (13). Reducing the significance level is appropriate when undertaking multiple comparisons, as is the case in this paper, to minimize the risk of reporting false significant differences due to chance (type 1 error) (14). Use of non-overlap of CIs in this manner is a simple and effective method to determine statistical significance in large surveys such as the one described in this paper. Note that unless otherwise stated, only those differences determined to be statistically significant using this method are discussed in Section “Results” of this paper.

## Results

A total of 2,500 individuals were surveyed between February and May 2014. A further 2,836 individuals did not wish to participate in the survey giving an overall response rate of 46.9%. As age was one of the variables used to weight the survey data, the 47 respondents who did not provide their age were excluded from the analysis, reducing the total sample size included in the statistical analysis to 2,453. The individuals excluded from the analysis on the basis that they did not give their age were spread evenly across all regions (Figure 1). Of this final survey sample, 952 individuals were male and 1,501 were female. Younger age groups were under-represented in this study (Table 1).

**Table 1:** **Demographic breakdown of survey respondents**.

Variables	Category	No of respondents	Proportion within each category (%)
Study sites	Gascoyne	196	8.0
	Geographe	198	8.1
	Goldfields-Esperance	197	8.0
	Great Southern	194	7.9
	Kimberley	191	7.8
	Leschenault	196	8.0
	Metropolitan Perth	297	12.1
	Midwest	196	8.0
	Peel	197	8.0
	Pilbara	194	7.9
	Southwest (other)	199	8.1
	Wheatbelt	198	8.1
Gender	Male	952	38.8
	Female	1,501	61.2
Age group (years)	18–34	199	7.8
	35–49	588	23.1
	50–64	854	33.6
	65+	812	31.9
Education	Primary school	31	1.3
	Lower secondary	516	21.0
	Upper secondary	656	26.7
	TAFE/college	508	20.7
	University	528	21.5
	Postgraduate	149	6.1
	Unsure	24	1.0
	Refused	41	1.7
Yearly income ($)	<50,000	687	28.0
	50,000–<100,000	540	22.0
	100,000–<150,000	368	15.0
	150,000–<200,000	181	7.4
	200,000–<250,000	69	2.8
	250,000+	54	2.2
	Unsure	146	6.0
	Refused	408	16.6

### Knowledge

Overall, the respondents exhibited a reasonable knowledge of RRV but poor knowledge of other mosquito-borne diseases in WA. A total of 68.5% of the survey population identified RRV as a locally acquired mosquito-borne disease within WA (Table 2). A total of 7.8 and 5.9% of respondents were able to identify MVE and BFV, respectively, while only 0.2% were able to identify WNV_KUN_ (Table 2). Twenty-one percent of respondents were unable to name a single mosquito-borne disease transmitted within WA, while others incorrectly identified diseases, such as malaria (17.8%) and dengue (15.7%), that are exotic to WA. Only a very small portion of respondents believed HIV to be transmitted by mosquitoes in WA (0.1%).

**Table 2:** **Knowledge of mosquitoes and mosquito-borne disease among respondents, with consideration given to region, gender and age group**.

	Region (%)												Age group (%)				Gender (%)		
Category	Kimberley	Pilbara	Gascoyne	Midwest	Goldfields Esperance	Wheatbelt	Perth Metro	Southwest (Peel)	Southwest (Leschenault)	Southwest (Geographe)	Southwest (other)	Great Southern	18–34	35–49	50–64	65+	Male	Female	State average (%)
**Correctly able to recognize the following as a locally acquired, mosquito-borne disease in WA**																			
RRV	85.4 (74.9–95.9)	76.9 (68.6–85.1)	69.8 (56.8–82.7)	71.7 (62.5–81.0)	79.9 (70.9–88.8)	81.8 (73.2–90.4)	63.1 (55.2–71.0)	82.7 (75.6–89.7)	83.8 (77.0–90.7)	82.5 (75.7–89.3)	83.2 (76.9–89.4)	77.0 (68.1–85.9)	*44.6 (30.8–58.4)	84.0 (76.6–91.4)	82.4 (76.9–87.9)	70.3 (64.3–76.3)	67.0 (58.5–75.5)	70.0 (62.2–77.7)	68.5 (62.7–74.2)
BFV	18.8 (11.6–26.0)	14.0 (8.1–19.8)	9.9 (5.2–14.7)	8.5 (3.4–13.5)	4.6 (0.9–8.3)	5.3 (2.1–8.5)	*2.5 (0.8–4.2)	19.8 (13.0–26.6)	17.5 (11.0–23.9)	13.6 (8.0–19.1)	9.4 (4.9–13.9)	8.3 (2.6–14.0)	*2.4 (0.6–4.2)	7.1 (3.3–10.9)	7.2 (4.5–10.0)	8.1 (5.1–11.1)	6.2 (3.8–8.5)	5.5 (3.7–7.4)	5.9 (4.4–7.4)
MVE	32.0 (20.5–43.6)	26.6 (18.8–34.4)	24.3 (12.8–35.7)	15.3 (8.8–21.9)	5.5 (1.3–9.6)	8.8 (5.1–12.5)	6.8 (3.4–10.2)	5.8 (2.2–9.4)	5.5 (2.6–8.5)	6.8 (3.2–10.3)	5.0 (2.1–7.8)	7.3 (3.6–11.0)	4.1 (0.0–9.3)	8.5 (3.8–13.2)	12.4 (7.8–16.9)	7.9 (4.5–11.3)	7.8 (4.7–10.8)	7.9 (4.1–11.6)	7.8 (5.4–10.2)
WNV_KUN_	2.5 (0.0–5.1)	2.8 (0.1–5.5)	–	0.4 (0.0–1.2)	–	–	–	–	–	–	–	1.6 (0.0–4.6)	0.1 (0.0–0.4)	0.4 (0.1–0.8)	0.0 (0.0–0.1)	–	0.2 (0.0–0.3)	0.1 (0.0–0.3)	0.2 (0.0–0.3)
**Do you know someone who has been diagnosed with rrv? (including self)**																			
Proportion (%)	81.0 (68.1–94.0)	66.7 (57.1–76.4)	67.2 (53.2–81.2)	61.6 (51.3–71.9)	73.3 (64.1–82.4)	64.9 (56.0–73.8)	48.8 (41.2–56.4)	70.8 (62.6–9.1)	72.7 (63.9–81.5)	73.9 (66.0–81.9)	69.8 (61.8–77.8)	64.9 (55.9–74.0)	44.9 (29.1–60.7)	56.5 (46.9–66.1)	60.5 (53.4–67.6)	61.3 (54.8–67.7)	49.3 (41.5–57.1)	62.4 (55.1–69.6)	55.8 (50.4–61.1)
**From which sources have you obtained information regarding mosquitoes and mosquito-borne disease?**																			
Health professional	30.9 (20.4–41.4)	31.1 (22.5–39.6)	29.2 (16.8–41.5)	30.6 (21.2–39.9)	20.4 (13.1–27.6)	15.7 (9.7–21.7)	19.9 (13.6–26.2)	30.3 (21.8–38.7)	24.3 (16.8–31.8)	15.2 (9.5–20.9)	22.5 (14.9–30.1)	22.1 (14.2–30.1)	24.9 (12.9–36.9)	19.4 (12.3–26.5)	26.9 (20.8–33.0)	14.4 (10.1–18.7)	20.6 (14.1–27.3)	22.7 (16.6–28.7)	21.7 (17.2–26.2)
Friend, family, and relative	52.9 (39.9–65.9)	50.6 (41.3–60.0)	52.2 (39.0–65.4)	55.4 (45.7–65.1)	60.8 (51.8–69.9)	46.3 (37.3–55.4)	47.7 (40.0–55.3)	54.6 (45.6–63.5)	62.6 (54.1–71.0)	49.9 (41.0–58.7)	59.5 (51.3–67.8)	54.3 (45.5–63.0)	58.5 (44.5–72.4)	51.6 (42.5–60.8)	44.9 (38.0–51.9)	40.0 (33.6–46.3)	48.8 (40.7–56.8)	50.9 (43.6–58.2)	49.8 (44.4–55.2)
Department of Health	37.2 (26.2–48.2)	33.7 (25.5–41.9)	24.7 (14.9–34.4)	30.3 (21.1–39.5)	27.6 (18.9–36.3)	27.9 (20.2–35.6)	15.1 (10.2–20.0)	36.0 (27.2–44.9)	35.1 (26.6–43.6)	26.3 (18.9–33.8)	27.1 (20.1–34.0)	31.2 (22.7–39.7)	14.0 (5.7–22.3)	21.5 (14.6–28.4)	26.8 (20.8–32.9)	21.5 (16.5–26.6)	24.6 (18.3–30.9)	16.0 (12.4–19.6)	20.3 (16.7–24.0)
Local government	54.4 (41.1–67.8)	54.8 (45.3–64.2)	41.0 (28.5–53.5)	32.0 (22.9–41.1)	22.4 (14.9–30.0)	28.9 (20.9–37.0)	14.5 (9.8–19.3)	43.9 (35.1–52.7)	42.3 (33.8–50.9)	47.4 (38.6–56.3)	27.4 (20.1–34.8)	30.6 (22.1–39.1)	13.9 (5.9–22.0)	23.5 (16.7–30.3)	29.5 (23.6–35.5)	24.8 (19.7–29.8)	21.0 (15.7–26.3)	23.2 (18.2–28.2)	22.1 (18.5–25.6)
Social media	16.2 (4.8–27.6)	28.4 (19.6–37.1)	14.0 (3.3–24.6)	18.8 (10.1–27.5)	26.5 (17.5–35.6)	12.0 (6.4–17.6)	14.7 (8.4–21.1)	27.9 (18.7–37.2)	18.4 (10.7–26.2)	15.1 (8.0–22.2)	21.1 (13.9–28.2)	16.3 (8.8–23.8)	30.1 (17.4–42.8)	16.1 (10.1–22.1)	10.9 (6.7–15.1)	*4.8 (2.3–7.3)	17.3 (10.4–24.1)	16.6 (10.8–22.4)	16.9 (12.4–21.4)
Internet (elsewhere)	30.8 (18.6–42.9)	29.6 (20.8–38.4)	17.2 (5.9–28.4)	27.3 (17.8–36.7)	22.9 (14.7–31.1)	15.9 (9.3–22.5)	23.8 (17.3–30.3)	33.0 (24.2–41.8)	23.0 (14.9–31.1)	29.8 (21.0–38.5)	20.4 (13.4–27.3)	18.6 (11.4–25.9)	25.8 (13.8–37.8)	30.5 (22.3–38.7)	27.1 (20.8–33.5)	*13.3 (8.7–17.9)	24.2 (17.5–31.0)	25.0 (18.8–31.3)	24.6 (20.0–29.3)
Print media	56.6 (43.2–70.1)	52.6 (43.2–62.0)	55.5 (42.2–68.8)	57.3 (47.7–66.9)	57.8 (48.0–67.5)	52.1 (42.9–61.3)	43.4 (35.9–50.9)	56.4 (47.3–65.4)	62.3 (53.5–71.0)	50.9 (41.9–59.8)	55.8 (47.1–64.4)	51.6 (42.7–60.5)	40.2 (26.4–54.0)	37.1 (28.8–45.5)	57.5 (50.5–64.4)	59.0 (52.5–65.4)	43.9 (36.1–51.7)	50.4 (43.2–57.7)	47.1 (41.8–52.5)
School, college, and university	21.6 (12.3–30.8)	21.0 (13.7–28.2)	12.4 (1.1–23.6)	8.5 (3.2–13.8)	13.3 (5.7–21.0)	6.5 (1.0–12.1)	11.0 (5.2–16.8)	12.6 (6.0–19.2)	7.2 (2.8–11.5)	11.7 (5.0–18.4)	13.4 (6.3–20.4)	14.1 (6.7–21.5)	22.0 (10.0–34.0)	6.8 (4.0–9.5)	6.8 (3.4–10.2)	6.6 (3.1–10.0)	13.1 (6.5–19.7)	9.8 (5.0–14.6)	11.5 (7.4–15.6)
Television	49.4 (36.3–62.4)	35.5 (27.1–43.8)	58.9 (46.1–71.7)	56.8 (47.3–66.3)	50.7 (40.8–60.6)	47.8 (38.7–56.9)	45.7 (38.2–53.2)	48.5 (39.5–57.4)	59.1 (50.2–68.1)	48.6 (39.7–57.4)	55.3 (46.8–63.9)	50.2 (41.3–59.2)	36.3 (22.8–49.8)	51.4 (42.3–60.6)	57.4 (50.5–64.4)	46.6 (40.1–53.1)	46.1 (38.2–54.0)	47.9 (40.7–55.1)	47.0 (41.6–52.3)
Radio	3.8 (0.8–6.8)	8.5 (1.9–15.2)	6.7 (0.0–14.6)	4.3 (1.0–7.5)	2.4 (0.0–4.7)	2.1 (0.1–4.2)	2.0 (0.2–3.8)	2.3 (0.0–4.7)	5.4 (1.0–9.9)	1.2 (0.0–2.7)	3.9 (1.1–6.7)	2.0 (0.2–3.8)	0.7 (0.0–1.4)	4.3 (0.0–8.6)	2.1 (0.3–3.9)	3.0 (0.8–5.1)	3.7 (1.2–6.2)	1.1 (0.3–2.0)	2.4 (1.1–3.7)
Work	0.9 (0.0–1.9)	13.4 (6.8–20.0)	0.9 (0.0–2.2)	3.7 (0.0–7.7)	6.5 (1.9–11.0)	0.6 (0.0–1.6)	1.2 (0.1–2.3)	1.7 (0.0–4.0)	1.5 (0.0–3.3)	0.3 (0.0–0.8)	1.5 (0.0–3.7)	–	0.6 (0.0–1.2)	2.5 (0.6–4.5)	3.2 (0.6–5.8)	0.8 (0.0–1.9)	2.3 (1.0–3.6)	1.1 (0.0–2.1)	1.7 (0.9–2.5)

*Cells shaded gray indicate that results are statistically higher than the state average*.

Cells shaded gray and marked with an * indicate that results are statistically lower than the state average.

Knowledge of mosquito-borne disease was clustered in two general regions of the state. Respondents from the southwest (Peel, Leschenault, and Geographe) and north of WA (Kimberley) were more knowledgeable on both RRV and BFV compared to the state as a whole (Table 2). Across WA, 55.8% of respondents had either been diagnosed themselves or knew someone who had been diagnosed with RRV. As expected, higher proportions of those diagnosed with the disease were clustered around higher risk regions in the Kimberley and the southwest, with the addition of the Goldfields-Esperance region (Table 2). It was not surprising to see that individuals from the Kimberley, Pilbara, and Gascoyne in northern WA had a greater awareness of MVE compared to the state as a whole. Knowledge of mosquito-borne disease differed significantly between age groups. Individuals aged 18–34 years were less able to correctly identify RRV and BFV than all older age groups. While there was an increasing likelihood of being diagnosed or knowing someone diagnosed with RRV as age increased, this was not considered a significant finding.

Overall, 74.8% (CI: 69.6–80.0) of the survey population believed that environmental conditions influenced the number of mosquitoes present. Hot weather after rainfall was the most common response (91.8%, CI: 87.8–95.8), followed by high tides (30.1%, CI: 24.9–35.4). Survey respondents most commonly identified stagnant water (60.9%, CI: 55.5–66.4), wetlands (38.5%, CI: 33.4–43.6), and domestic containers (20.8%, CI: 17.0–24.7) as regular mosquito breeding sites. While there were few regional differences, it is interesting to note that individuals from the Peel region were more aware of the role that wetlands (57.5%, CI: 48.5–66.6) and tidal activity (45.6%, CI: 35.9–55.3) play in mosquito breeding compared to the state as a whole.

Information regarding mosquitoes and mosquito-borne disease was most commonly sourced from family and friends (49.8%) followed by traditional print media (47.1%) and television (47.0%) (Table 2). Local and state governments played a greater role in informing the public in the Kimberley, Pilbara, and south-west regions, but played a limited role in the Perth metropolitan region. The survey demonstrated the increasing role that social media (16.9%) and the Internet (24.6%) are now playing in information delivery. As expected, these sources are significantly more important to individuals aged 18–34 years than those aged over 50 years. Older individuals aged over 65 years relied most heavily on print media (Table 2). Respondents in the Pilbara region were statistically more likely to have obtained information on mosquitoes and mosquito-borne disease through their workplace than all other regions (Table 2).

### Attitudes

Across WA, 24.5% of survey respondents felt mosquitoes posed a health risk where they lived, 42.9% felt they were a nuisance, while 32.6% felt they were of no concern at all (Table 3). Regional differences were evident in attitudes held toward mosquitoes and the impact they had on the lives of the individual surveyed. Respondents from the Pilbara, Goldfields/Esperance, and Leschenault regions were most concerned with the health risk posed by mosquitoes compared to the state average, as were individuals from the Kimberley, although the small cross over in CIs deemed this an insignificant finding. Those living in the Perth metropolitan area were one of the least likely regions to be concerned with health risks associated with mosquitoes but most concerned with the nuisance problem (Table 3). Individuals aged 65+ years were least concerned with the impact of mosquitoes. While not a statistically significant finding, there was trend for younger individuals aged 18–34 years to be least concerned with mosquitoes as a health risk and more concerned about the nuisance factor.

**Table 3:** **Attitudes of respondents in regards to mosquitoes and mosquito-borne disease, with consideration given to region, gender, and age group**.

	Region												Age group				Gender		
Category	Kimberley	Pilbara	Gascoyne	Midwest	Goldfields Esperance	Wheatbelt	Perth Metro	Southwest (Peel)	Southwest (Leschenault)	Southwest (Geographe)	Southwest (Other)	Great Southern	18–34	35–49	50–64	65+	Male	Female	Total
**What impact do mosquitoes have on your quality of life?**																			
Health risk	37.7 (26.6–48.8)	37.4 (28.8–46.0)	30.7 (18.9–42.5)	22.5 (15.6–29.3)	39.9 (30.0–49.8)	19.5 (13.8–25.2)	21.2 (15.3–27.1)	36.1 (27.9–44.3)	40.3 (31.5–49.2)	26.4 (19.2–33.6)	29.0 (21.4–36.5)	19.4 (13.3–25.4)	17.7 (7.3–28.1)	28.3 (20.6–36.1)	27.4 (21.5–33.2)	26.9 (21.1–32.7)	22.7 (16.1–29.3)	26.3 (20.9–31.7)	24.5 (20.3–28.8)
Nuisance	35.9 (22.6–49.1)	38.7 (29.3–48.1)	37.1 (23.6–50.6)	33.1 (23.8–42.4)	43.5 (33.6–53.4)	44.7 (35.5–53.9)	45.7 (38.0–53.4)	35.7 (26.8–44.6)	29.9 (21.3–38.6)	41.9 (33.0–50.9)	26.8 (19.4–34.1)	36.4 (27.3–45.4)	51.5 (37.4–65.6)	48.7 (39.5–57.9)	41.8 (34.7–48.8)	*24.5 (19.0–30.0)	39.4 (31.6–47.3)	46.4 (39.0–53.8)	42.9 (37.4–48.4)
No concern	26.4 (13.2–39.7)	23.9 (15.6–32.1)	32.2 (20.3–44.1)	44.4 (34.7–54.1)	16.6 (11.4–21.8)	35.8 (26.9–44.6)	33.1 (26.0–40.1)	28.2 (19.9–36.6)	29.8 (22.2–37.3)	31.7 (23.5–39.8)	44.3 (35.8–52.8)	44.3 (35.5–53.1)	30.8 (17.7–43.9)	23.0 (15.0–30.9)	30.9 (24.4–37.4)	48.6 (42.1–55.1)	37.8 (30.1–45.6)	27.3 (21.1–33.5)	32.6 (27.6–37.6)
**During the worst times of the year, how often do you get bitten by mosquitoes?**																			
Everyday	45.9 (32.8–58.9)	23.5 (15.9–31.1)	23.1 (11.7–34.6)	12.2 (6.5–17.9)	27.6 (18.4–36.9)	26.8 (19.2–34.4)	16.0 (10.2–21.8)	30.6 (22.8–38.4)	19.7 (13.1–26.3)	21.3 (13.2–29.3)	15.5 (9.3–21.8)	17.1 (9.7–24.4)	19.4 (8.4–30.3)	24.6 (17.2–32.0)	17.9 (13.1–22.7)	11.4 (7.6–15.2)	14.8 (9.9–19.7)	22.6 (16.1–29.1)	18.7 (14.6–22.8)
**In which locations are you bitten by mosquitoes?**																			
Home	83.7 (72.8–94.6)	66.3 (57.2–75.4)	84.8 (75.7–93.9)	73.0 (63.6–82.3)	79.9 (71.6–88.1)	81.7 (73.2–90.2)	74.7 (66.7–82.7)	88.6 (81.8–95.5)	83.5 (76.1–90.9)	84.2 (76.8–91.7)	81.0 (73.7–88.3)	74.8 (65.9–83.7)	64.7 (50.2–79.3)	82.8 (75.3–90.3)	82.5 (76.9–88.0)	82.8 (77.1–88.5)	72.2 (63.5–81.0)	81.7 (75.1–88.3)	76.9 (71.3–82.5)
Recreation	66.0 (54.0–77.9)	75.3 (67.2–83.4)	50.6 (36.6–64.6)	45.5 (35.4–55.7)	57.4 (47.4–67.5)	41.6 (31.9–51.3)	47.5 (39.0–56.0)	48.5 (39.2–57.8)	44.0 (34.7–53.4)	42.9 (32.8–52.9)	43.6 (34.2–53.0)	54.7(45.2–64.3)	60.3 (45.7–74.8)	50.6 (41.1–60.0)	42.1 (34.9–49.3)	*33.2(26.2–40.1)	49.0 (40.3–57.7)	48.3(40.5–56.1)	48.6(42.8–54.5)
Work	30.1 (19.6–40.5)	31.2 (22.7–39.7)	24.4 (12.2–36.5)	18.2 (9.0–27.4)	34.6 (24.2–44.9)	17.8 (10.9–24.7)	4.9 (1.5–8.3)	14.0 (8.6–19.4)	6.2 (1.6–10.7)	10.0 (5.0–15.1)	11.0 (4.6–17.4)	10.2 (4.6–15.9)	7.3 (1.3–13.4)	10.4 (6.6–14.2)	13.5 (8.9–18.1)	4.1 (1.6–6.7)	7.9 (5.5–10.3)	10.0 (5.6–14.3)	8.9 (6.4–11.4)
**How much of a problem do you think mosquitoes are where you live? (1 – not a problem; 4 – significant problem)**																			
Rating	3.0 (2.8–3.2)	2.6 (2.4–2.7)	2.2 (2.1–2.4)	*2.1 (1.9–2.2)	2.6 (2.5–2.8)	2.4 (2.2–2.6)	2.2 (2.1–2.3)	2.7 (2.5–2.8)	2.5 (2.3–2.6)	2.3 (2.2–2.5)	*2.1 (2.0–2.2)	*2.0 (1.9–2.1)	2.2 (2.1–2.4)	2.4 (2.3–2.6)	2.3 (2.2–2.4)	*2.1 (2.0–2.2)	2.1 (2.0–2.3)	2.4 (2.3–2.5)	2.3 (2.2–2.3)
**How concerned are you about catching a mosquito-borne disease? (1 – not concerned; 5 – very concerned)**																			
Rating	3.5 (3.1–3.9)	3.0 (2.8–3.3)	3.1 (2.8–3.4)	3.0 (2.8–3.3)	3.3 (3.0–3.5)	3.0 (2.7–3.2)	2.8 (2.6–3.0)	3.4 (3.2–3.6)	3.2 (3.0–3.5)	3.0 (2.8–3.2)	3.0 (2.8–3.2)	2.8 (2.6–3.0)	2.8 (2.4–3.2)	2.8 (2.6–3.1)	3.1 (2.9–3.2)	2.9 (2.8–3.1)	*2.5 (2.3–2.7)	3.3 (3.2–3.5)	2.9 (2.8–3.0)

*Cells shaded gray indicate that results are statistically higher than the state average*.

Cells shaded gray and marked with an * indicate that results are statistically lower than the state average.

Individuals from the Kimberley, Pilbara, Goldfields-Esperance, and Southwest (Peel and Geographe) all rated the mosquito problem in their area as being worse than the state average (Table 3). The same areas, with the exception of the Pilbara, were also more concerned about the risk of acquiring a mosquito-borne disease. Conversely, individuals from the Midwest, Southwest (other), and Great Southern rated their mosquito problem as being less severe than the state average. Respondents aged 35–49 years were more concerned with the mosquito problem in their area compared to the state average, while those aged 65+ years were least concerned. There were no statistical age differences in the level of concern in regards to mosquito-borne disease. Interestingly, females rated the mosquito problem in their region and the likelihood of acquiring a mosquito-borne disease to be more concerning than males.

Individuals from the Kimberley (45.9%) and Peel (30.6%) regions were more likely to be bitten by mosquitoes on a daily basis during the worst times of year, compared to the state as a whole (18.7%) (Table 3). There was no statistical difference in the reported frequency of mosquito bites between males and females. Older individuals (65+ years) were more likely to report that they are not bitten at all (24.8%, CI: 19.0–30.6) compared to the state average (13.1%, CI: 9.6–16.6). The majority of individuals across WA were bitten at home (76.9%), while 48.6% reported being bitten during recreational activities and 8.9% at work. Regional differences also existed in the location in which respondents received mosquito bites (Table 3). Individuals from the Kimberley (30.1%), Pilbara (31.2%), Gascoyne (24.4%), and Goldfields-Esperance (34.6%) were more likely to be bitten at work than the state as a whole (8.9%), while individuals in the Pilbara were more likely to be bitten during outdoor recreational activities (75.3%) compared to the state as a whole (48.6%). Outdoor recreational activities also appeared to be a risk factor for mosquito bites in the Kimberley, but this finding was not considered significant due to a small cross over in the CIs. Individuals aged 65+ years were less likely to be bitten by mosquitoes during recreational activities than the state average.

### Practices

The most common methods respondents used to reduce mosquitoes on their property included killing mosquitoes as they noticed them (85.6%) and eliminating stagnant water and/or water holding containers (71.9%) (Table 4). A total of 33.2% of individuals sprayed residual insecticide on their property, a practice most commonly adopted by individuals within the Peel region. Interestingly, residents in the Kimberley and Southwest (Leschenault) were less likely to use a residual spray than the state average (Table 4). Other methods of mosquito reduction included changing pet water at least weekly, maintaining pools adequately, stocking ponds with fish, and maintaining rainwater tanks; however, not all households owned pets or had these amenities, making it difficult to report.

**Table 4:** **Practices in regards to mosquito avoidance among respondents, with consideration given to region, gender, and age group**.

	Region												Age group				Gender		
Category	Kimberley	Pilbara	Gascoyne	Midwest	Goldfields Esperance	Wheatbelt	Perth	Southwest (Peel)	Southwest (Leschenault)	Southwest (Geographe)	Southwest (Other)	Great Southern	18–34	35–49	50–64	65+	Male	Female	Total
**Which measures do you take to reduce the number of mosquitoes present on your property?**																			
Kill as noticed	96.7 (94.3–99.1)	93.5 (89.8–97.2)	92.9 (89.0–96.7)	81.9 (74.3–89.5)	94.0 (90.8–97.3)	86.7 (79.6–93.7)	84.3 (79.0–89.7)	88.1 (81.1–95.0)	89.2 (82.8–95.7)	85.4 (79.2–91.6)	86.2 (80.7–91.6)	85.3 (79.0–91.5)	87.7 (78.6–96.7)	83.1 (75.4–90.7)	87.6 (82.7–92.4)	83.6 (78.6–88.5)	83.8 (78.2–89.3)	87.4 (82.2–92.6)	85.6 (81.8–89.4)
Residual spray	*17.1 (10.0–24.1)	24.9 (17.9–31.8)	27.0 (15.4–38.6)	25.6 (17.5–33.8)	28.3 (19.2–37.5)	32.8 (24.6–41.0)	34.0 (26.6–41.4)	45.2 (36.2–54.2)	24.4 (17.0–31.9)	18.9 (12.7–25.1)	25.2 (18.0–32.5)	26.2 (18.1–34.4)	38.6 (24.6–52.6)	33.2 (24.6–41.7)	28.3 (22.2–34.5)	30.5 (24.5–36.6)	28.8 (21.2–36.4)	37.7 (30.5–50.0)	33.2 (28.0–36.5)
Eliminate stagnant water	88.0 (77.5–98.5)	81.7 (73.7–89.6)	78.8 (67.4–90.2)	76.4 (67.8–85.1)	84.7 (78.3–91.1)	72.0 (62.8–81.2)	69.4 (62.2–76.6)	74.3 (66.2–82.4)	88.3 (83.9–92.7)	77.1 (69.7–84.5)	75.9 (67.9–84.0)	67.1 (58.2–76.1)	67.2 (53.7–80.7)	74.3 (65.9–82.7)	76.2 (70.0–82.4)	71.5 (65.5–77.4)	67.2 (59.3–75.2)	76.6 (70.4–82.8)	71.9 (66.8–77.0)
**What measures have you taken in the last 12 months to protect yourself and family from being bitten?**																			
Mosquito coils	76.8 (66.0–87.6)	68.9 (60.2–77.5)	54.5 (41.2–67.8)	46.8 (37.3–56.4)	64.6 (55.9–73.2)	54.6 (45.5–63.8)	44.6 (37.0–52.1)	52.1 (43.2–61.1)	54.6 (45.8–63.4)	42.2 (33.5–50.9)	51.9 (43.4–60.4)	40.0 (31.3–48.7)	44.3 (30.4–58.2)	51.9 (42.7–61.0)	56.8 (49.9–63.8)	37.3 (31.0–43.6)	44.6 (36.7–52.5)	50.4 (43.1–57.7)	47.5 (42.1–52.9)
Repellent	87.3 (77.3–97.2)	85.3 (78.2–92.5)	73.0 (60.0–85.9)	72.6 (64.4–80.9)	90.3 (86.4–94.2)	72.4 (63.8–81.0)	76.5 (70.2–82.9)	85.3 (79.2–91.4)	81.9 (74.4–89.3)	75.3 (67.3–83.3)	70.1 (61.9–78.4)	69.0 (60.9–77.1)	78.1 (66.2–90.0)	86.1 (79.4–92.8)	77.1 (71.1–83.1)	67.5 (61.4–73.6)	75.4 (68.7–82.2)	79.9 (73.9–85.9)	77.7 (72.2–82.2)
Clothing	67.6 (54.6–80.7)	75.1 (66.8–83.3)	56.7 (43.6–69.8)	62.9 (53.6–72.2)	69.8 (60.0–79.6)	63.7 (54.5–72.8)	56.8 (49.2–64.3)	61.7 (52.8–70.5)	68.0 (59.6–76.5)	62.6 (54.1–71.0)	68.4 (60.2–76.6)	68.3 (60.2–76.4)	58.8 (44.9–72.8)	59.4 (50.1–68.6)	62.7 (55.9–69.6)	57.1 (50.7–63.6)	54.6 (46.6–62.6)	64.4 (57.4–71.5)	59.5 (50.1–64.8)
Stay indoors	76.4 (65.2–87.5)	73.6 (64.9–82.2)	64.3 (52.2–76.4)	55.1 (45.3–64.9)	66.2 (56.7–75.6)	60.2 (51.0–69.4)	62.9 (55.5–70.4)	74.2 (65.9–82.5)	74.0 (65.8–82.2)	76.5 (69.7–83.3)	55.9 (47.3–64.5)	61.1 (52.6–69.6)	62.7 (49.0–76.5)	66.8 (57.9–75.7)	68.7 (62.2–75.3)	60.8 (54.4–67.1)	56.9 (48.9–64.8)	72.6 (65.9–79.2)	64.7 (59.4–69.9)
Insect screens	94.4 (89.7–99.1)	93.4 (87.8–99.0)	97.4 (95.1–99.7)	93.9 (89.2–98.6)	95.9 (93.0–98.7)	93.7 (89.1–98.3)	95.0 (91.9–98.1)	94.5 (89.5–99.6)	98.0 (96.0–100.0)	92.9 (87.3–98.4)	90.2 (85.5–94.9)	93.3 (90.1–96.6)	95.0 (89.6–100.0)	96.3 (92.1–100.6)	94.7 (91.6–97.8)	92.7 (89.3–96.2)	93.8 (90.1–96.8)	95.8 (92.3–99.2)	94.8 (92.5–97.0)
Operate fans	87.6 (80.7–94.4)	62.0 (52.8–71.2)	72.9 (60.6–85.2)	61.9 (52.6–71.3)	*40.7 (30.9–50.5)	52.6 (43.4–61.9)	50.0 (42.3–57.6)	55.3 (46.3–64.3)	61.9 (53.4–70.5)	*47.3 (38.5–56.0)	*45.1 (36.7–53.4)	*27.5 (19.5–35.6)	50.6 (36.4–64.7)	*47.6 (38.5–56.7)	55.2 (48.2–62.2)	52.6 (46.1–59.1)	*47.5 (39.6–55.5)	55.0 (47.7–62.2)	51.2 (58.8–56.6)
Electronic zapper	11.2 (5.4–16.9)	17.4 (10.5–24.3)	13.7 (4.4–22.9)	22.4 (14.8–30.0)	27.6 (18.9–36.2)	19.3 (12.9–25.6)	24.1 (17.2–31.0)	29.3 (21.0–37.6)	25.8 (17.9–33.7)	18.6 (11.9–25.2)	24.3 (16.3–32.3)	21.2 (13.4–29.1)	28.8 (15.7–41.8)	25.6 (17.4–33.8)	22.6 (16.9–28.3)	16.6 (11.9–21.2)	23.9 (16.6–31.2)	24.1 (17.6–30.6)	24.0 (19.1–28.9)
Automatic spray	25.9 (14.6–37.3)	38.6 (29.2–48.0)	31.7 (18.7–44.6)	31.3 (21.7–40.9)	34.8 (25.0–44.5)	23.6 (16.3–30.8)	27.2 (20.3–34.1)	35.6 (26.8–44.4)	29.2 (21.0–37.3)	23.7 (16.7–30.8)	29.4 (21.9–36.8)	32.6 (23.5–41.7)	30.7 (17.9–43.4)	32.8 (24.0–41.5)	22.5 (16.9–28.1)	26.8 (21.0–32.6)	30.0 (22.2–37.7)	27.3 (21.4–33.1)	28.6 (23.7–33.5)
Mosquito netting	35.7 (21.6–49.8)	24.1 (16.2–32.1)	17.6 (8.7–26.4)	14.3 (8.3–20.3)	18.1 (10.5–25.8)	12.6 (7.9–17.3)	8.7 (4.0–13.4)	9.3 (4.8–13.9)	14.7 (7.9–21.4)	13.6 (6.9–20.2)	18.2 (11.3–25.1)	18.5 (10.7–26.3)	13.2 (4.0–22.3)	10.6 (5.4–15.7)	10.3 (6.6–14.1)	7.9 (4.7–11.0)	10.8 (5.6–16.1)	10.6 (6.7–14.6)	10.7 (7.4–14.1)
Other	8.5 (0.0–18.4)	4.9 (0.4–9.3)	3.2 (0.6–5.8)	11.6 (5.7–17.5)	5.9 (1.9–9.8)	4.8 (1.9–7.6)	4.3 (1.3–7.3)	2.4 (0.7–4.1)	2.5 (0.4–4.5)	5.1 (1.9–8.3)	2.0 (0.3–3.7)	1.5 (0.0–3.1)	3.7 (0.0–9.2)	4.0 (1.0–6.9)	4.8 (1.9–7.6)	5.0 (2.0–8.1)	3.8 (2.7–7.4)	4.7 (2.6–6.9)	4.3 (2.2–6.4)
None of the above	–	0.2 (0.0–0.7)	0.4 (0.0–1.2)	3.1 (0.0–6.4)	0.2 (0.0–0.6)	3.3 (0.0–7.5)	1.5 (0.0–2.9)	–	0.6 (0.0–1.6)	0.9 (0.0–2.2)	0.8 (0.0–1.9)	3.0 (0.8–5.2)	0.3 (0.0–0.8)	1.6 (0.0–4.6)	2.1 (0.0–4.3)	1.8 (0.1–3.5)	2.1 (1.5–4.0)	0.5 (0.0–1.2)	1.3 (0.3–2.3)
**Which mosquito repellent have you used in the past 12 months?**																			
Chemical-based repellent	92.8 (87.4–98.3)	93.7 (87.3–100.0)	89.2 (83.7–94.7)	95.6 (93.0–98.2)	95.4 (91.7–99.1)	94.0 (90.4–97.5)	93.2 (89.2–97.3)	92.8 (88.9–96.7)	94.3 (89.1–99.5)	91.2 (85.7–96.8)	94.0 (90.1–97.9)	93.7 (89.2–98.3)	95.2 (88.1–102.3)	92.1 (87.3–96.8)	94.4 (91.0–97.8)	91.0 (86.5–95.4)	95.0 (90.3–99.6)	91.8 (88.4–95.0)	93.3 (90.5–96.2)
Repellent wipes/towelette	6.1 (2.4–9.9)	10.6 (4.6–16.6)	6.3 (2.2–10.5)	4.1 (1.3–6.8)	15.2 (8.0–22.4)	11.4 (6.1–16.7)	10.0 (4.9–15.1)	6.8 (3.4–10.2)	7.5 (2.3–12.6)	11.1 (5.2–17.1)	7.0 (3.0–10.9)	17.1 (8.2–26.1)	8.8 (0.0–18.0)	6.8 (2.0–11.6)	13.4 (7.9–18.9)	11.4 (6.5–16.2)	6.2 (3.1–9.3)	13.0 (6.9–19.0)	9.7 (6.2–13.2)
Repellent bracelet	5.2 (1.6–8.9)	9.2 (4.7–13.8)	3.2 (0.6–5.9)	10.3 (2.6–18.0)	20.1 (11.1–29.1)	11.4 (3.5–19.3)	8.5 (3.6–13.5)	19.1 (10.8–27.4)	10.3 (4.6–15.9)	12.9 (6.1–19.7)	5.9 (1.5–10.3)	6.6 (2.7–10.5)	12.1 (2.3–21.8)	8.5 (4.4–12.6)	10.0 (5.6–14.5)	9.2 (4.3–14.0)	8.2 (3.2–13.3)	11.8 (6.8–16.7)	10.0 (6.5–13.6)
Citronella-based repellent	64.7 (51.9–77.4)	57.2 (47.5–66.8)	48.0 (33.5–62.5)	44.6 (33.4–55.7)	60.9 (50.3–71.5)	59.1 (49.2–69.0)	51.4 (42.6–60.1)	47.1 (37.3–56.8)	54.7 (45.0–64.5)	45.6 (35.6–55.7)	61.8 (52.3–71.3)	46.5 (35.6–57.3)	53.4 (37.5–69.2)	57.3 (47.6–66.9)	53.3 (45.4–61.2)	38.4 (30.8–46.0)	48.5 (39.2–57.8)	54.7 (46.8–62.7)	51.7 (45.6–57.8)
Other natural-based repellent	26.5 (16.2–36.9)	29.0 (20.0–38.0)	11.1 (5.3–16.8)	19.6 (11.3–27.9)	13.6 (6.2–21.0)	10.5 (5.8–15.1)	8.8 (3.7–13.9)	11.4 (5.8–17.0)	10.2 (4.3–16.0)	6.1 (2.6–9.6)	16.5 (9.6–23.4)	13.7 (7.2–20.1)	10.8 (1.2–20.3)	14.3 (8.2–20.5)	9.6 (5.9–13.4)	5.7 (2.4–9.1)	7.1 (2.1–12.2)	13.9 (8.8–19.1)	10.6 (7.0–14.2)
Other	1.0 (0.0–2.2)	3.1 (0.5–5.7)	1.6 (0.0–3.5)	1.4 (0.0–2.7)	1.0 (0.0–2.2)	1.6 (0.0–3.5)	0.5 (0.0–1.2)	1.8 (0.2–3.5)	2.1 (0.3–4.0)	1.0 (0.0–2.5)	6.4 (2.0–10.8)	1.9 (–0.3–4.0)	0.1 (–0.1–0.2)	0.6 (0.2–1.1)	1.8 (0.2–3.4)	2.1 (0.1–4.1)	0.4 (0.2–0.6)	1.5 (0.5–2.5)	0.9 (0.4–1.5)

*Cells shaded gray indicate that results are statistically higher than the state average*.

Cells shaded gray and marked with an *indicate that results are statistically lower than the state average.

Use of insect screens was the most frequently utilized method to protect against being bitten by mosquitoes across all regions in WA (94.8%) (Table 4); however, regional variation existed in other common measures. Mosquito netting and mosquito coils were used more frequently in northern WA, including the Kimberley and Pilbara regions. Individuals from the Pilbara were also more likely to utilize long-sleeved clothing. Respondents from the Goldfields-Esperance respondents were more likely to apply insect repellent than the state as a whole. Of those who reported using mosquito repellent in the past 12 months, the majority (93.3%) had used a chemical-based formulation. However, just over half the respondents (51.7%) also reported using citronella-based products. Use of other repellent products, such bracelets or wipes, accounted for only 10.0 and 9.7% of respondents, respectively. Overall, a similar proportion (10.6%) reported using other natural-based mosquito repellents. Interestingly, the proportion of respondents from the Kimberley (26.5%) and Pilbara (29.0%) who used natural-based products was significantly higher than most other regions of WA and the State as a whole.

Only 29 (1.3%) respondents claimed that they did not use any form of personal protection against mosquitoes. The majority (24/29) felt this was because they did not notice mosquitoes in their area and the remainder stated they were not worried about being bitten. No respondents claimed that it was because of a dislike for chemical use.

## Discussion

The results of this survey indicate that the majority of respondents are aware of the potential for mosquitoes in WA to transmit RRV. While this is encouraging, there is still work to be done to ensure WA residents further develop their knowledge of mosquito-borne disease, methods of reducing mosquito breeding on their property, and simple ways to practice mosquito avoidance. There is an immediate need to increase awareness of arboviruses other than RRV in Australia, as well as the significance of exotic mosquito-borne diseases to travelers heading overseas. The lack of awareness surrounding MVE is a particular concern, given the potentially fatal nature of the disease. In 2010–2011, 16 cases of MVE were reported in Australia, 9 of which were in WA (including 1 death) (15). During this time, the Department of Health issued four media statements following continued detection of MVE in sentinel chicken flocks and increasing numbers of human cases of disease (16). This information was further publicized at a regional and local level by population health units and local governments (16). Despite these and more recent efforts to increase awareness of MVE, only 7.8% of survey respondents listed MVE as a locally transmitted mosquito-borne disease. An even smaller proportion of survey respondents were aware of WNV_KUN_. Given only five cases of this disease have occurred in WA since 2000, with the last case reported in 2006, this was perhaps an expected result.

Unsurprisingly, individuals surveyed from the northern regions of the state (Kimberley, Pilbara, and Gascoyne) were more aware of MVE and WNV_KUN_, which reflects the regional distribution of the virus. However, the persistently low proportion of respondents aware of these diseases highlights the immediate need for a more effective communication strategy regarding MVE and WNV_KUN_ virus disease risks targeting both residents and travelers to northern WA. While it is a concern that knowledge of BFV remains limited, the distribution of the virus is similar to RRV throughout the state and is associated with less severe clinical symptoms (5). If individuals are taking precautions to prevent RRV, then they are effectively also protecting themselves against BFV.

Interestingly, malaria and dengue were listed as the second and third most frequent mosquito-borne diseases transmitted in WA after RRV. While both are significant mosquito-borne diseases overseas, local transmission does not occur in WA as the vectors have not established here. The increased awareness of malaria and dengue among WA residents is likely to be due to the global re-emergence of both diseases (1). Despite the absence of local transmission, 62 cases of malaria and 535 cases of dengue were notified to the WA Department of Health in 2013/2014 (Department of Health, unpublished data). All notified malaria cases and all except one dengue case were reported in travelers who acquired the infection overseas and were diagnosed after returning to WA (Department of Health, unpublished data). The first case of dengue believed to be locally acquired in WA for over 70 years was notified in October 2013 from Point Sampson in the Pilbara region (17). This was thought to have resulted from the temporary incursion of an infected dengue vector mosquito that failed to establish a local population (17). The majority of the overseas acquired dengue cases were from individuals who traveled to Bali and Indonesia, and the majority of cases of malaria were among travelers to, and refugees from, African countries (Department of Health, unpublished data). Future communication efforts need to focus on educating WA residents traveling overseas of the risks associated with mosquitoes and simple, effective ways to prevent being bitten.

### Regional Variation

The regional variation in knowledge, attitudes, and practices observed in this study largely reflects the geographic variation in mosquito-borne disease incidence throughout WA. The Kimberley not only experiences the highest incidence rate of RRV notifications in most years, but historically, it also reports the highest number of MVE cases (15). Almost half of all survey respondents from the Kimberley reported being bitten by mosquitoes on a daily basis. Knowledge of mosquitoes and mosquito-borne disease among respondents from the Kimberley was generally more accurate than the state as a whole and concern surrounding the possibility of acquiring a mosquito-borne disease was higher. Despite these findings, future communication efforts in the region are still warranted to raise awareness of the significance of MVE and to educate residents on the importance of preventing backyard mosquito breeding. Communication efforts also need to target younger age groups who were identified in the survey as being less knowledgeable and less concerned with the health risks associated with mosquitoes.

In contrast, individuals from the Perth Metropolitan, Midwest, Southwest (elsewhere), and the Great Southern regions all rated their perceived mosquito nuisance problems as being less severe than the State as a whole. This was an expected result, particularly for Perth, as it reports the lowest incidence rate of RRV compared to all other regions. Respondents from the Perth region were significantly less knowledgeable on mosquito-borne diseases, less likely to be diagnosed themselves or know someone diagnosed with RRV, and less likely to receive information from either state or local government on this topic, when compared to the majority of other regions surveyed. While the risk of acquiring a mosquito-borne disease in Perth may be lower than in other regions, it is still important for individuals to be aware of the potential for disease transmission. Residents living in low risk areas are likely to travel to other regions in WA or to overseas destinations where the risk of acquiring a mosquito-borne disease is significantly higher. Given there is no specific treatment or cure for locally acquired mosquito-borne diseases, prevention through education is the only effective way to reduce the impact these viruses have on the individual and the healthcare system.

Significant regional differences existed in the location (home, work, and recreation) in which respondents received mosquito bites. The increased likelihood of being bitten at work in the Kimberley, Pilbara, Gascoyne, and Goldfields-Esperance is an important finding in this study. This finding is likely attributed to the greater proportion of outdoor workers employed in the energy, mining, and resources industry in these regions. Prior to 2012, very few cases of RRV were reported each year in the Goldfields-Esperance region. Following significant rainfall in late February 2013, more than 200 notified cases of RRV were acquired in this region over the following 2 months. This may explain why respondents from this region were more concerned about the mosquito problem in their area and the likelihood of acquiring a mosquito-borne disease than the State as a whole. Case follow-up data revealed that a common site of exposure for many infected individuals was their workplace (Department of Health, unpublished data). This presents a potential occupational health and safety risk that needs to be addressed by employers. It is encouraging to see that some proactive employers within this sector actively educate their employees on the risks associated with mosquitoes as part of their induction and/or ongoing employee training particularly in the Pilbara region where respondents were significantly more likely to report obtaining information regarding mosquitoes and mosquito-borne disease from their workplace.

The significantly increased likelihood of being bitten during a recreational activity in the Pilbara likely reflects the warmer weather in northern WA and an increased appreciation for outdoor recreational activities at dawn and dusk when mosquitoes are more likely to be active. Individuals undertaking outdoor recreational activities in the northern regions of WA, including outdoors sports, gardening, fishing, caravanning and camping, are therefore considered to be at increased risk of acquiring a mosquito-borne disease. Significant efforts need to be undertaken to raise awareness among these “at risk” groups, particularly in younger age groups who remained less aware of the potential health impact posed by mosquitoes.

The high rate of mosquito bites occurring at home across all regions is concerning. In explaining this result, it must be acknowledged that regional variation in mosquito ecology/population abundance and urban planning exists. In some regions, the high proportion of individuals being bitten at home may be largely attributed to the close proximity of housing to local wetlands, as has been demonstrated in the Peel, Geographe and Leschenault regions of WA (18–20). In other regions, particularly those in the Perth Metropolitan area, it is important to consider the potential role of domestic container breeding within the urban environment. The disparity between the high proportion of respondents who reported being bitten at home (76.9%) and the small proportion of respondents who identified the importance of domestic containers as a potential mosquito breeding habitat (20.8%) is a concern. While local and state government work collaboratively to reduce mosquito breeding in the natural environment, it is important that future communication efforts focus on educating people on simple ways to reduce potential mosquito breeding on their own property.

### Age Variation

Knowledge of mosquito-borne disease was least developed in the youngest age group included in this study. While younger individuals were under-sampled in this study, data were weighted by age to the 2013 estimated resident population (12) to reduce the impact of selection bias on the results. Significantly fewer individuals in the youngest age group (18–34 years) were able to correctly identify mosquito-borne diseases endemic to WA and were less likely to have experienced RRV themselves or to have known someone with RRV. These findings may partially explain why younger respondents consider mosquitoes to be more of a nuisance than a health risk.

Reduced concern for health and participation in risky behavior is common among adolescents and young adults. This is demonstrated by their increased likelihood to participate in activities, such as binge drinking and illicit substance use (21). Interestingly, individuals in this age bracket did not report being bitten more regularly than older age groups included in the survey and their prevention practices were also comparable. In this instance, it appears that knowledge and attitudes may not necessarily reflect behavior or prevention practices. This finding may be due to a number of reasons. Individuals in the younger age group may still apply repellent at a similar rate to older age groups despite their lowered level of concern, simply to avoid the inconvenience of being bitten by mosquitoes. Respondents were asked to list the prevention practices they had used in the past 12 months. It is possible that individuals in the youngest age bracket used repellent less often, yet answered yes to this question because they have used the prevention practice at least once during this time. Recall bias is also a possible threat to the validity of this study, as individuals needed to recollect information from the past 12 months. An alternative explanation is that younger individuals spend less time at home where the majority of bites among respondents were reported. While not a statistical finding, the results demonstrate an increasing likelihood of being bitten at home as age increased. Exposure to mosquitoes around the home may occur more often in older individuals participating in outdoor activities such as gardening or entertaining. The increasing trend for younger individuals to be bitten during recreational activities suggests that exposure to mosquitoes differs among age groups.

The results of this survey suggest that younger individuals may be considered an “at risk” group for mosquito-borne disease, due to their reduced awareness and concern for the risks associated with mosquitoes. Despite their apparent good use of prevention practices, future communication efforts need to be targeted at those aged 18–34 years to improve knowledge of mosquito ecology and mosquito-borne disease. Recent reports demonstrate that the majority of individuals acquiring dengue overseas and returning to Australia are young and middle-aged adults (22). While this may reflect the frequency of travel among these age groups to south-east Asian destinations (22), it is also possible that a reduced awareness of the risks associated with mosquitoes combined with an increased likelihood of participation in risky behavior are contributing factors.

### Gender Variation

Female respondents were significantly more concerned about the mosquito problem in their area and the likelihood of acquiring a mosquito-borne disease compared to males. This increased level of concern may be partially attributed to more women reporting to know individuals diagnosed with RRV than their male counterparts. While this latter trend was not considered statistically significant, the CI cross over was marginal, suggesting that with a larger sample size it is likely to have been a significant finding. Individuals who have had a personal experience with the debilitating disease may naturally be more concerned about the risk of disease transmission. While females were more likely to move indoors to avoid mosquitoes compared to male, there were no statistical differences in the knowledge males and females held in regards to mosquitoes, mosquito-borne disease, or the frequency that they were being bitten.

### Study Limitations

The potential for sampling bias should be noted as a limitation of this study. Despite best efforts to obtain a representative sample of survey respondents, younger age groups and males were under-represented in the study. Furthermore, regional areas outside of Perth were deliberately oversampled in order to generate valid estimates for each and to make comparisons between regions. As noted previously, the survey results were weighted by age, sex, and region against the 2013 estimated resident population to adjust for this sampling bias and obtain representative estimates for each demographic group within the WA population (12). Recall bias may also be present in this study. In various questions, respondents were asked to recall information from the 12 months prior. Inaccurate information may have been provided by study participants as a result of poor recollections retrieved from the past. This is particularly relevant with regard to practices used to prevent mosquito bites and situations where individuals were bitten by mosquitoes.

### Importance of a Targeted and Sustainable Communications Campaign

The need to reduce the incidence of mosquito-borne disease in WA is twofold. The Department of Health has an obligation to protect, promote, and improve the health of the public of WA, and to reduce the incidence of preventable illness. Given the significantly debilitating impact of mosquito-borne diseases in WA caused by RRV, BFV, WNV_KUN_, and the potentially fatal MVE, together with the absence of either a vaccine or cure for any of these diseases, there is an even greater obligation for the Department to actively promote disease prevention.

A reduction in disease incidence will also reduce the substantial cost associated with clinical infection. A conservative cost estimate for RRV disease in Australia, calculated in 2001, was estimated to range between A$2.7 and A$5.6 million annually (3). This excludes the cost of other investigations to rule out differential diagnoses, alternative treatments, decreased work output beyond 1 week, and the intangible costs associated with pain and suffering (3). The true cost is likely to be significantly higher. To justify and design preventive programs, it is important to consider the economic costs of disease and weigh this up against the costs of promoting prevention.

Sustainability of mosquito-borne disease prevention is essential. Although some short-term victories are necessary, greater emphasis should be placed on programs anticipated to have a sustained positive public health impact (23). The Department of Health will use these results to inform the development of a long term, all-encompassing communication campaign to educate the general public on mosquitoes and mosquito-borne disease.

An innovative communication campaign will be developed and rolled out over the 2015–2016 arboviral season in WA to actively raise awareness of mosquito-borne disease among the general public and “at risk” groups identified through this survey. A toolkit of resources will be developed to support and complement existing communication efforts by the local government. Newer communication channels, such as social media, will be explored to reach younger age groups identified as lacking knowledge in this area. The new communication strategy will be described and rigorously evaluated in a future paper by repeating the KAP survey to determine if knowledge, attitude, and practices relating to mosquitoes and mosquito-borne disease in WA have improved.

## Conclusion

The results of this survey indicate that the majority of respondents are aware of the potential for mosquitoes in WA to transmit RRV, but awareness of other endemic mosquito-borne diseases was limited. The survey also highlighted the existence of limited awareness of the potential for backyard breeding in domestic containers, occupational exposure to mosquitoes particularly in regions with a large employment base in the mining sector, increased exposure to mosquitoes during recreational activities in the north of the State, and reduced awareness of mosquito-borne disease in individuals aged 18–34 years. The results of this survey will be used to develop a new communication strategy in WA by the Department of Health to reduce the impact of mosquito-borne disease.

## Author Contributions

AP: wrote the KAP survey, liaised with SRC to conduct survey, wrote draft copy of manuscript, and submitted. AJ: input into KAP survey development, analyzed data, and reviewed manuscript. PJN: project managed the survey and reviewed manuscript.

## Conflict of Interest Statement

The authors declare that the research was conducted in the absence of any commercial or financial relationships that could be construed as a potential conflict of interest.
